# Murine and Chinese cobra venom-derived nerve growth factor stimulate chondrogenic differentiation of BMSCs *in vitro*: A comparative study

**DOI:** 10.3892/mmr.2021.12444

**Published:** 2021-09-16

**Authors:** Zhikang Miao, Zhenhui Lu, Shixing Luo, Danqing Lei, Yi He, Huayu Wu, Jinmin Zhao, Li Zheng

Mol Med Rep 18: 3341-3349, 2018; DOI: 10.3892/mmr.2018.9307

Subsequently to the publication of this paper, an interested reader drew to the authors’ attention that Figs. 2 and 3, showing the results from experiments designed to assess the viability of bone-derived mesenchymal stem cells culture with or without nerve growth factors (NGFs) via fluorescein diacetate/propidium iodide or H&E staining respectively, contained apparently duplicated data panels within the figures. After having examined their original data, the authors have realized that these figures were inadvertently assembled incorrectly, and that there were also misassembled data panels in Fig. 6, which showed the secretion of types I and II collagens in bone-derived mesenchymal stem cells with or without NGFs.

The corrected versions of Figs. 2, 3 and 6 are shown below and on the next page. Note that these errors did not significantly affect the results or the conclusions reported in this paper, and all the authors agree to this Corrigendum. Furthermore, the authors apologize to the readership for any inconvenience caused.

## Figures and Tables

**Figure 2.**
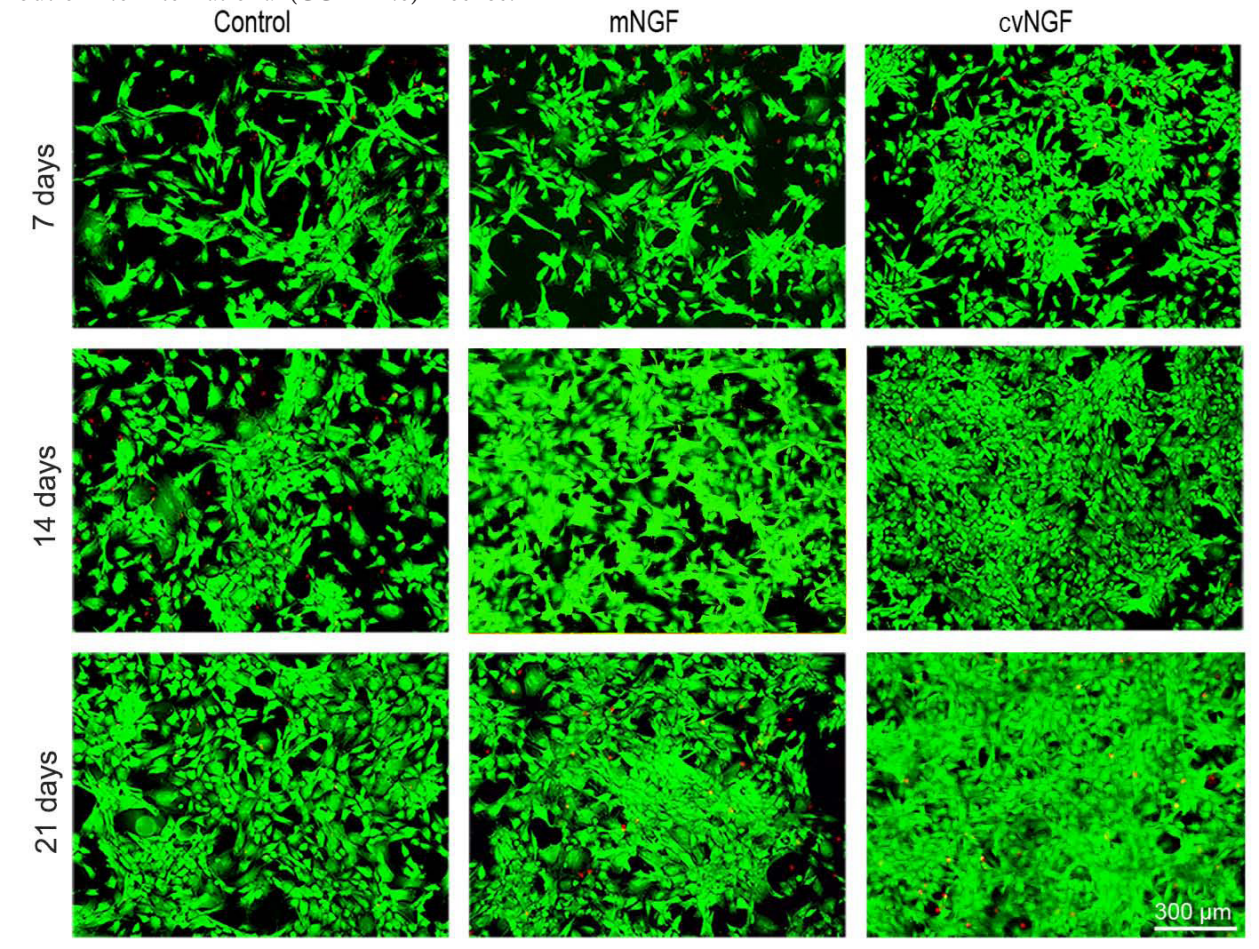
Fluorescein diacetate/propidium iodide staining was used to detect the viability of bone-derived mesenchymal stem cells cultured alone (control) or with NGFs (mNGF, 0.06 µg/ml; cvNGF, 6 µg/ml) for 7, 14 and 21 days. Cell seeding density, 2×10^4^/ml (original magnification, ×100; scale bar, 300 µm). cvNGF, cobra-venom-derived NGF; mNGF, murine β-NGF; NGF, nerve growth factor.

**Figure 3.**
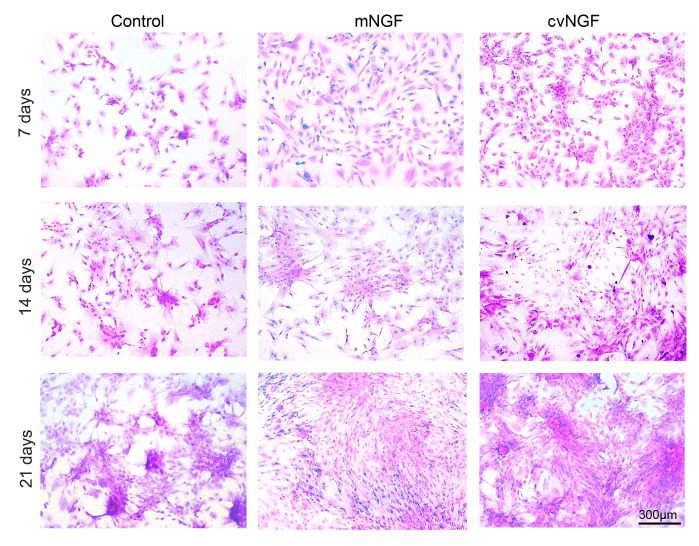
Hematoxylin and eosin staining was used to detect the morphology of bone-derived mesenchymal stem cells cultured alone (control) or with NGFs (mNGF, 0.06 µg/ml; cvNGF, 6 µg/ml) for 7, 14 and 21 days. Cell seeding density, 2×10^4^/ml (original magnification, ×100; scale bar, 300 µm). cvNGF, cobra-venom-derived NGF; mNGF, murine β-NGF; NGF, nerve growth factor.

**Figure 6.**
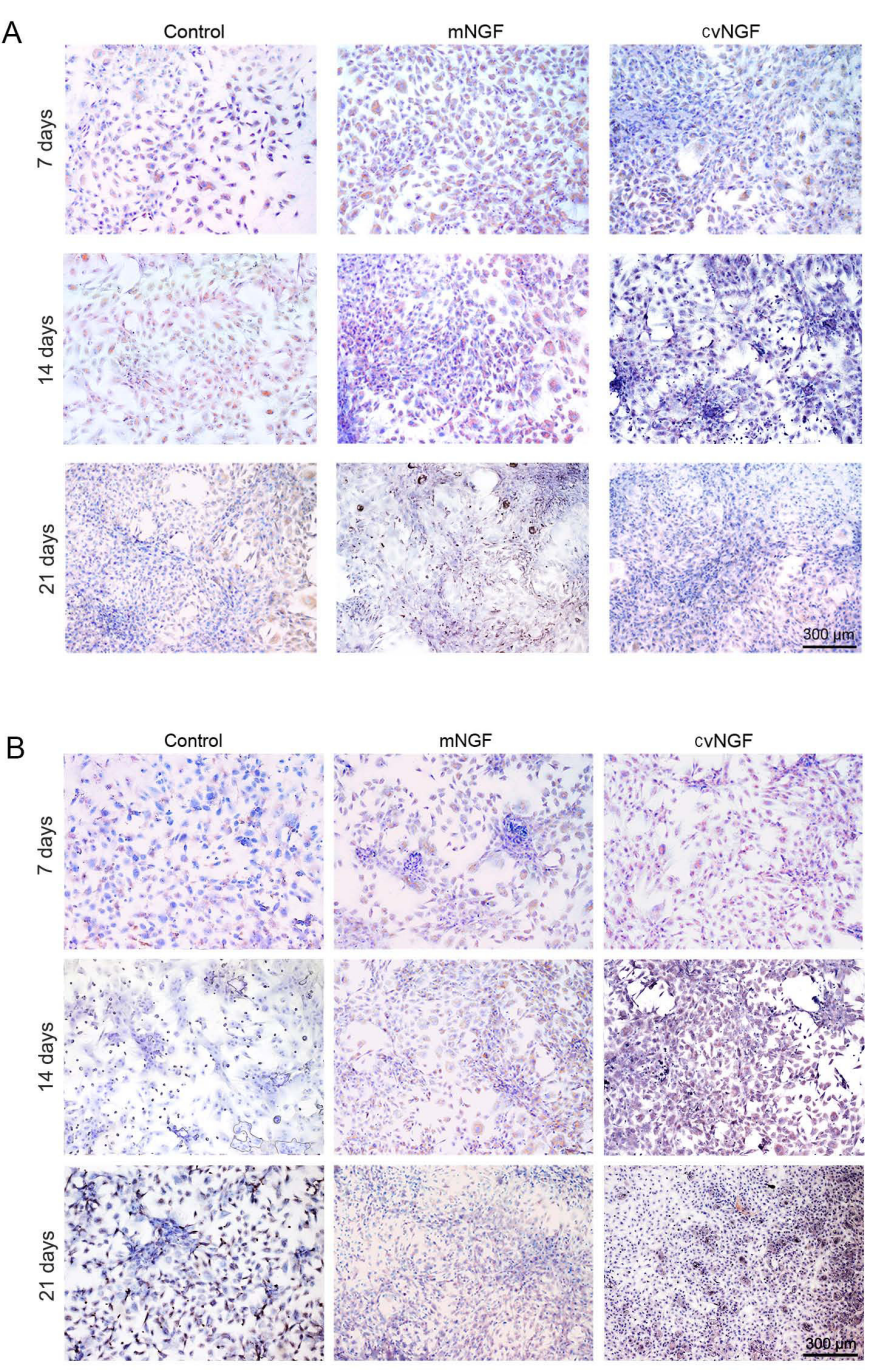
Immunohistochemical staining was used to detect the secretion of (A) type I and (B) type II collagens in bone-derived mesenchymal stem cells cultured alone (control) or with NGFs (mNGF, 0.06 µg/ml; cvNGF, 6 µg/ml) for 7, 14 and 21 days. Cell seeding density, 2×10^4^/ml (original magnification, ×100; scale bar, 300 µm). cvNGF, cobra-venom-derived NGF; mNGF, murine β-NGF; NGF, nerve growth factor.

